# Gut microbial community structure of the adult citrus root weevil *Diaprepes abbreviatus*

**DOI:** 10.3389/finsc.2025.1676003

**Published:** 2025-10-28

**Authors:** Imilce A. Rodriguez-Fernandez, Tasha M. Santiago-Rodriguez, Paola G. Figueroa-Pratts, Keislamarí Cintrón-Berríos, Nichole D. Rodriguez-Cornier, Gary A. Toranzos

**Affiliations:** ^1^ Microbiology and Genetics Laboratory, Department of Biology, University of Puerto Rico, San Juan, Puerto Rico; ^2^ The Alkek Center for Metagenomics and Microbiome Research, Department of Molecular Virology and Microbiology, Baylor College of Medicine, Houston, TX, United States; ^3^ Department of Molecular Virology and Microbiology, Baylor College of Medicine, Houston, TX, United States; ^4^ Environmental Microbiology Laboratory, Department of Biology, University of Puerto Rico, San Juan, Puerto Rico

**Keywords:** *Diaprepes abbreviatus*, coleoptera, gut anatomy, gut microbiome, metagenomic sequencing, gut pH, citrus pest

## Abstract

*Diaprepes abbreviatus* is an agricultural pest known to affect around 270 plant species across the Caribbean and the United States, posing significant challenges to pest management. Chemical control dominates management, but environmental and health concerns motivate microbiome-informed alternatives. However, limited information exists on the gut anatomy, physicochemical environment, and microbial composition of *D. abbreviatus.* In this study, we provide the first comprehensive characterization of the gut morphology, pH, and microbiota of adult *D. abbreviatus* in both females and males collected in Puerto Rico. Using dye-based gut tracing, we identified foregut, midgut, and hindgut or posterior gut compartments, and confirmed the presence of a muscular, sclerotized gizzard. Colorimetric analysis revealed a mildly acidic gut environment (approximately pH 4–5, based on qualitative ranges), consistent across sexes and regions. Shotgun metagenomic sequencing of dissected guts from males and females revealed microbial communities distinct from the leaf samples microbiota. While alpha and beta diversity did not differ significantly between sexes, co-occurrence analyses identified sex-specific correlation patterns among bacterial taxa. Notably, *Enterobacter cloacae*, *Pantoea vagans*, *Lactococcus lactis*, and *Pseudomonas monteilii* were repeatedly detected across individuals and generated metagenomic datasets, and some were localized to the hindgut, suggesting possible niche specialization. The presence of taxa, such as *Enterobacter cloacae*, previously reported as symbionts in other phytophagous insects further supports the hypothesis that certain bacteria may contribute to host digestion or adaptation. These findings establish a framework for understanding the gut environment and microbial community of *D. abbreviatus*, and highlight candidate taxa for future functional studies. More broadly, this work supports further research into the potential roles of gut microbiota in the ecology and management of this pest.

## 1 Introduction

The citrus root weevil (*Diaprepes abbreviatus*, Linnaeus) is a highly destructive beetle species in the Curculionidae family, native to the Caribbean, including the island of Puerto Rico. It is historically known that this polyphagous pest feeds on around 270 plant species including ornamental, sugarcane, vegetables, and especially citrus (1–3). *Diaprepes abbreviatus* has been found in several areas of the continental United States, including Florida (4), California (5, 6), Texas (7) and Louisiana (State of Louisiana. Department of Agriculture and Forestry, 2009). It was likely introduced accidentally to a nursery in Florida in 1964 through shipments of infested ornamental plants from Puerto Rico (8). Since then, its establishment in Florida has caused significant economic losses to the citrus industry with damages estimated at approximately 70 million dollars annually (8).

The ecological impact of *D. abbreviatus* extends beyond superficial damage, as the insect attacks different plant roots during its developmental stages. Larvae, which remain at this stage for 8–15 months, feed on fibrous and structural roots creating wounds that may facilitate colonization by soil-borne pathogens (e.g., *Phytophthora* spp.), leading to root rot, decline, and eventual plant death (6, 9). Adults, which can live on average 147 days (females), and 135 days (males), feed on young, tender leaves, reducing the plant’s ability to produce energy through photosynthesis (6). Although adults rarely feed on fruit, it is typically limited to papaya and citrus (6). The weevil’s invasive nature and resilience have challenged agricultural communities, rendering traditional control methods, including cultural practices, biological interventions, and chemical treatments, largely ineffective (10–13). In 2023, *D. abbreviatus* was identified by the EFSA (European Food Safety Authority) Panel on Plant Health as a relevant non‐regulated European Union (EU) pest that could potentially enter the EU (14). Because *D. abbreviatus* is primarily spread via the movement of infested plants and soil, its associated microbiome may contribute to survival during transport and establishment in new environments, representing an overlooked factor in invasion pathways.

Recent evidence suggests that the interaction between herbivorous insects and their host plants is mediated by the gut microbiome, which refers to all the microorganisms (i.e., bacteria, fungi, viruses, archaea) that reside within the gut. Studies have linked the gut microbiome to key insect physiological and biological functions, including energy provision, nutrient uptake, growth, degradation of plant toxins and pesticides, and shaping of the immune system (reviewed in 15, 16). These insect–microbiota interactions can influence plant–insect relationships both directly and indirectly. For example, certain gut bacteria mediate host plant selection by influencing insect preferences and aiding in the identification of suitable plants for feeding and reproduction. Once on the plant, insects encounter challenges such as low nutritional quality, indigestible plant tissues, and toxic secondary metabolites. Gut bacteria help overcome these obstacles by enhancing digestion, supplying essential nutrients, and detoxifying harmful compounds, allowing insects to exploit otherwise unsuitable hosts. Conversely, plant defenses or poor-quality plant tissue can disrupt the gut microbiota (dysbiosis), and may negatively impact insect health, development, and survival [reviewed in (17)].

Several examples illustrate the abovementioned microbial contributions. For instance, termites such as *Macrotermes gilvus* utilize gut bacteria such as *Providencia* spp. and *Bacillus* spp. to produce cellulase, which degrades cellulose (18); *Streptomyces* and *Pantoea* species provide cellulase to the wood-eating wasp *(* 19 *)*; and in the Coleoptera order, species such as *Cyrtotrachelus buqueti* and *Osphranteria coerulescens* rely on *Bacillus velezensis* and *Bacillus safensis* for cellulase production, while *Holotrichia parallela* utilizes *Pseudomonas* spp. for the same purpose (20–22).

Studies using 16S rRNA gene amplicon sequencing have shown that the gut bacterial composition of insects is influenced by developmental stage, diet, sex, and environmental factors (23–26). These findings have encouraged researchers to investigate the potential role of gut bacteria in insect health and physiology as a potential target for innovative pest control strategies (16, 17). Despite this progress, little is known about the adult *D. abbreviatus* gut microbiome or how it is influenced by sex, diet and other environmental factors. To our knowledge, most research to date has focused primarily on its biology, behavior, and population control (6, 8, 14), with its genome sequenced only recently in 2024 (27).

Our research group has previously analyzed the fecal bacteria of *D. abbreviatus* fed three different diets, namely lemon (*Citrus aurantifolia*), guava (*Psidium guajava*), and passion fruit (*Passiflora edulis*), using 16S rRNA gene amplicon sequencing, and found that the gut bacterial composition varied depending on the insect’s diet (28). Based on that study, we proposed that specific gut bacteria may aid in breaking down the unique secondary metabolites present in different host plants. However, our previous study did not identify potential gut-specific and transient microbes (i.e., those acquired through diet) in association with gut regions and sex. Gut regions in insects vary in pH and redox state, and not only influence digestive enzyme activity (29, 30), but can also create complex microenvironments that may support specific microbial communities (15). This has previously been described for the alimentary canal of adult weevils in the family Curculionidae, which comprise distinct regions (e.g., foregut, midgut, and hindgut), each with unique morphology at both macroscopic and microscopic levels (31–37). Similarly, sex-based differences in the gut microbiota have also been reported across several insect groups, often reflecting underlying physiological, hormonal, or behavioral differences between males and females. For instance, in *Anopheles gambiae* mosquitoes, males and females harbor distinct microbial profiles that are linked to differences in sugar- versus blood-feeding (38). Sex-specific microbiome shifts have also been associated with mating status and reproductive investment in *Drosophila melanogaster* (39). Differences in pH have previously been noted in female mosquitoes under different feeding conditions (40). However, to our knowledge, no studies have directly compared gut pH between male and female insects, representing an important gap in understanding the physiological factors shaping sex-specific gut environments. These studies show an association between the microbiome, diet and sex, with potential consequences for host fitness and ecological interactions.

Here, we aim to describe the gut morphology, determine regional pH, and characterize the gut microbiota of adult male and female *D. abbreviatus*, providing the first detailed analysis of this pest’s gut structure and microbial communities. We combined anatomical characterization, colorimetric pH measurements, and shotgun metagenomic sequencing to profile bacterial communities at the species level across different gut regions and between sexes. These results not only expand our understanding of *D. abbreviatus* physiology, but also offer new insights into the potential role of the microbiome in this insect’s polyphagous capacity. To the best of our knowledge, these findings represent the first comprehensive examination of gut anatomy and microbial community structure in adult male and female *D. abbreviatus*. This knowledge could be leveraged to develop future innovative pest management strategies by targeting or modifying the microbiome.

## 2 Materials and methods

### 2.1 Insect collection and handling

Adult *D. abbreviatus* are large, oval-shaped insects measuring 10–19 mm in length, with a distinctive black and tan/orange color pattern on their elytra (wing covers) (1, 6, 8). The larvae are legless with curved, cylindrical bodies and reddish-brown heads (1). Despite polymorphism, all individuals exhibit black and tan/orange patches (41). Insects in this study were collected directly from lemon trees at Finca La Tres Once, Florida, Puerto Rico (18.3722° N, 66.5328° W). Collections were made in summer 2022, September–October 2024, and April 2025, corresponding to the experiments described below. Unless otherwise specified, insects were transported live in ventilated containers for immediate treatment and dissections, or stored in ethanol for downstream analyses (see Section 2.4).

### 2.2 Gut dissection, anatomical characterization and imaging

Gut anatomy experiments were performed on insects collected in summer 2022. To study the gut anatomy of the insect, we used visual inspection and dye-based tracking. Various insects were fed 2% (w/v) Blue dye No. 1 (Erioglaucine disodium salt, CAS 3844-45-9; Fisher Scientific, Waltham, MA, USA; Cat. No. 18-602-610), suspended in 1% agarose (CAS 9012-36-6; Sigma Cat. No. A-5093). This dye has been successfully used in studies with the fruit fly *D. melanogaster*, as it is nontoxic and not absorbed by the intestinal epithelium remaining in the gut lumen (42, 43). This Blue dye helped confirm complete gut ingestion and dissection. Gut regions were anatomically defined based on gross morphology and insect gut schematics (reviewed in 15). We designated the foregut, midgut, and hindgut, with the proventriculus serving as a boundary between the foregut and midgut. Images were taken by adapting a Pixel6a (Google) or an iPhone 14 (Apple) smartphone to the ocular of a Nikon Stereo microscope.

To further characterize gut structures at the microscopic level, we prepared dissected tissues for scanning electron microscopy (SEM) imaging (see **Supplementary Methods** for details). Using this protocol, we were able to obtain images of the proventriculus, while other tissues proved to be more challenging.

### 2.3 Gut pH measurement

Gut pH experiments were conducted on insects collected in September–October 2024. To assess gut pH across different regions, we designed a colorimetric assay using multiple pH indicators. Feeding with 2% Bromophenol Blue in 5% sucrose has been used to evaluate regional pH changes, with the acidic middle midgut staining yellow and the rest of the midgut blue in *D. melanogaster* (44).

Live insects collected from Finca La Tres Once were brought to the laboratory, sexed, and randomly assigned to one of three experimental groups: 24h–24h, 48h–24h, and 72h–24h. In this scheme, the first number indicates the duration of the fasting period, and the second the duration of feeding with the pH dye-containing agarose prior to dissection. Each pH indicator was prepared in a 1% agarose (CAS 9012-36-6; Sigma Cat. No. A-5093) solution in deionized water (unbuffered) with a final dye concentration of 2% (w/v). The indicators used were Thymol Blue (CAS 76-61-9; Fisher Scientific Cat. No. T-416; transition ranges: 1.2–2.8, red to yellow, and 8.0–9.6, yellow to blue), Bromophenol Blue (CAS 34725-61-6; Sigma Cat. No. B6131; transition range: 3.0–4.6, yellow to blue), and Bromophenol Red (CAS 2800-80-8; Carolina Biological Supply Company; transition range: 3.0–4.6, yellow to blue), allowing us to cover a broad pH spectrum across gut regions. This colorimetric assay provides qualitative ranges of gut pH rather than precise quantitative measurements, and results are interpreted accordingly.

After the feeding period, insects were dissected under a Nikon stereomicroscope, and images were taken using an iPhone 14 Pro Max (Apple) or iPhone 12 (Apple) adapted to the microscope ocular. The color of the gut contents was compared to standard reference charts for each dye to determine pH (40, 45, 46). The 24-hour and 72-hour fasting groups included two to three biological replicates, with 2–3 insects collected per replicate. The 48-hour fasting group experiment was performed only once, with one male and one female for the Bromophenol Blue and Bromophenol Red assays, and two females for the Thymol Blue assay. Gut content colors were scored independently by three observers through visual inspection.

### 2.4 Insect collection and dissection for metagenomics

Full-gut metagenomic analyses were performed on insects collected in April 2025. In the same farm, insects were collected aseptically using gloves and sterile 50 mL Falcon tubes pre-filled with 40 mL of 99% Molecular grade ethanol (CAS 64-17-5; Sigma Cat. No. E7023) (4–5 insects per tube), and kept on ice during transport to the lab. Environmental data (temperature, humidity, barometric pressure, elevation, and coordinates) were recorded at the time of collection. To assess potential microbial overlap, a pool of ~30 lemon tree leaves were also collected from the same trees using sterile 50 mL Falcon tubes (sample 1 containing: 2.13 g and sample 2 containing 3.83g of dried leaves). Following collection, insects were stored in 99% ethanol at 4 °C for no more than 24 hours to prevent tissue brittleness. Insect weight and size were recorded prior to dissection, but were not considered critical parameters (28).

In the laboratory, insects underwent a surface sterilization protocol to remove external microbial contaminants. This included two washes in sterile distilled water to remove ethanol, two sequential immersions in 1% commercial bleach (Clorox Bleach, The Clorox Company, sodium hypochlorite), followed by two additional washes in sterile, DNA-free water (VWR Cat. No. 10220-382). Sterilized insects were transferred to sterile Petri dishes containing sterile 1× PBS (Fisher Cat. No. BP399500) for dissection under a dissection microscope. Sex was determined after dissection.

Using sterile scalpels and forceps, the head, legs, wings, and exoskeleton were carefully removed to expose internal organs including the gut. The anterior, mid, and posterior gut sections were isolated aseptically and placed in sterile 1.5 mL microcentrifuge tubes containing 1 mL sterile 1× PBS on ice. PBS was removed, and guts were flash-frozen on dry ice and stored at –80 °C until further processing. DNA extraction and shotgun metagenomic sequencing were performed by Zymo Research (Irvine, CA, USA) (described below).

Two lemon tree leaf composite samples (~30 leaves per sample) were collected at the moment of collecting the insects to determine any potential taxa that could be originating from the insect, or the leaf. The leaves were dried at the moment of sending the samples to Zymo for sequencing. Given the limited amount of leaf sample replicates included in this study, results would be for illustrative purposes and no statistical inferences can be made.

### 2.5 DNA extraction, library preparation and sequencing

We outsourced Zymo Research (Irvine, CA, USA) sequencing services, where DNA was extracted from full guts using the ZymoBIOMICS^®^-96 MagBead DNA kit, following manufacturer’s instructions. Libraries were then prepared using the Illumina^®^ DNA Library Prep Kit (Illumina, San Diego, CA) following manufacturer’s instructions using unique dual-index 10 bp barcodes with Nextera^®^ adapters (Illumina, San Diego, CA) and paired, 2 x 150bp reads. Libraries were then pooled in equal abundance, and the final pool was quantified using qPCR and TapeStation^®^ (Agilent Technologies, Santa Clara, CA), and sequenced using NovaSeq^®^ X (Illumina, San Diego, CA). The ZymoBIOMICS^®^ Microbial Community Standard (Zymo Research, Irvine, CA) was used as a positive control for each DNA extraction, and for each library preparation. Negative controls, including a blank extraction control and a blank library preparation control, were added to assess cross-contamination and bioburden.

### 2.6 Bioinformatic analyses

Raw sequence processing and annotation were performed at Zymo Research. Reads were trimmed to remove low quality fractions and adapters using Trimmomatic-0.33 (47). Briefly, quality trimming by sliding window with 6 bp window size and a quality cutoff of 20 was used, and reads with size smaller than 70 bp were removed. Low-diversity reads were removed using sdust (https://github.com/lh3/sdust), and microbial composition was determined using Sourmash (48). The Genome Taxonomy Database (GTDB) species representative database (RS207) was used for bacterial and archaeal identification. Pre-formatted GenBank databases (v.2022.03) provided by Sourmash (https://sourmash.readthedocs.io/en/latest/databases.html) were also used for fungi identification. Reads were mapped back to the genomes identified by Sourmash using BWA-MEM (49), and the microbial abundance was determined based on the counts of mapped reads (50). The number of raw reads, the percentage of reads remaining after trimming, and low diversity reads for these data are shown **Supplementary Table 1**. Species were called if more than 10 reads aligned to its genome and had relative abundances greater than 0.0001%.

### 2.7 Diversity analyses

Alpha diversity analyses were performed in RStudio (v.2024.04.2 + 764) using the phyloseq package (v.1.48.0). The number of observed features and Shannon diversity index were determined using the estimate_richness function. Since relative abundance tables provided by Zymo were used as input for diversity analyses, no rarefaction was performed (see **Supplementary Table 2**). Alpha diversity results were then visualized through boxplots generated using the ggplot2 package (v.3.5.1) in RStudio. Statistical significance was assessed through Mann-Whitney analysis with p-value correction using the False Discovery Rate (FDR) algorithm by Benjamini & Hochberg (51). Beta diversity analyses were performed using ATIMA (Agile Toolkit for Incisive Microbial Analyses) (https://atima.research.bcm.edu/), a stand-alone tool for analyzing and visualizing microbiome datasets. Beta diversity analyses were performed using the R function vegan:adonis version 2.5.5 to estimate Bray-Curtis distances, which were then visualized through Principal Coordinate Analysis (PCoA) plots, and significance was assessed through Permutational Analysis of Variance (PERMANOVA) and using 999 permutations.

### 2.8 Taxonomic analysis

The relative abundances of the top 15 most abundant bacterial species were determined using ATIMA, and visualized through stacked bar plots for female, male and leaf samples. Venn diagrams were constructed using the VennDiagram (v.1.7.3) package in RStudio to visualize the number of unique and shared taxa across female, male and leaf samples. Additional Venn diagrams were done using Venny 2.1 (52). Spearman co-correlation analyses of bacterial species in female and male samples were performed using the corr.test() function from the psych package (v.2.4.12) in RStudio. Significant rho values after FDR p-value adjustment (correlation matrix) were visualized through correlograms generated using the corrplot package (v.0.95). No inclusion or filtering criteria was applied for the Venn-diagrams and correlograms due to the low number of insects included in this study and since several taxa were insect-specific (see Results).

### 2.9 Collection, sequencing and annotation methods comparison

Our initial shotgun metagenomics experiments aimed to explore gut microbiota differences across gut regions and sexes in *D. abbreviatus*. The genomic DNA of samples were extracted in-house and sent to Novogene for sequencing (see **Supplementary Methods**). However, we noted contamination from human skin microbiota when performing the analyses, and taxonomic profiles were dominated by *Cutibacterium acnes*, as well as taxa from the genus *Staphylococcus*. Several aspects of sample collection may have affected results. For instance, insects were collected in the wild without gloves, and surface sterilization was performed using 70% ethanol, and two washes with sterile PBS (see **Supplementary Methods**). This protocol seems to have been insufficient to completely eliminate human-associated bacteria. To address this, we curated the dataset by identifying bacterial species known to be common on human skin and excluded or filtered them from subsequent analyses (see for the list of removed taxa and for the curated data **Supplementary Tables 3**, **4**).

We then repeated insect collection using enhanced precautions as described above in Section 2.4, with DNA extraction and library preparation performed by Zymo Research. These samples did not exhibit detectable human skin bacterial contamination, supporting the validity of the filtering criteria applied to the main dataset in the study, also referred to as the ‘Gut regions Shotgun Metagenomics’ dataset, and indicating that the remaining taxa reflected the insect-associated microbiota. These findings also confirmed the effectiveness of the improved collection and sterilization procedures in minimizing human-associated contamination. We also compared results at the genus level with previous 16S rRNA gene V4 amplicon data (28), to identify potential genera shared across various sequencing approaches, and annotation tools (see **Supplementary Methods**).

## 3 Results

### 3.1 Gut anatomy

**Figure 1:**
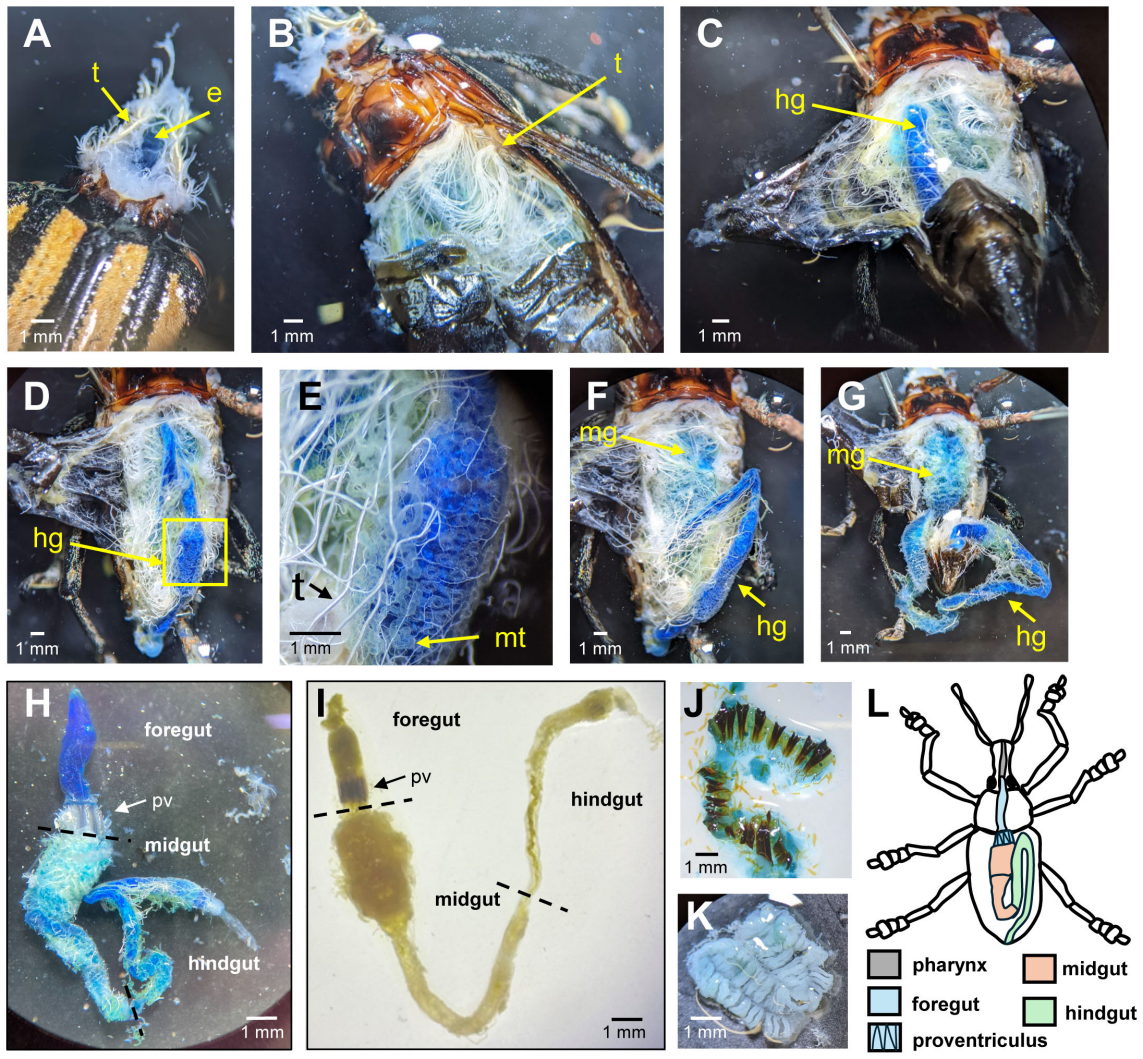
Adult *D. abbreviatus* gut anatomy. To visualize gut compartments, an adult *D. abbreviatus* was fed 2% blue dye in 1% agarose. Panels **(A–H, J, K)** show images taken at different points during dissection. In panels **(A–C)** the tracheal system (t) is visible as white filamentous structures surrounding the gut. The esophagus (e) is also noted in panel **(A)**. In panel **(D–G)**, the gut is visualized as it was stained with blue dye, facilitating the differentiation of the foregut (fg), midgut (mg), and hindgut (hg). Panel **(E)** provides a close-up view of the gut region highlighted in yellow in panel **(D)**, where Malpighian tubules (mt) appear as thin, thread-like structures surrounding the midgut-hindgut junction. Panels **(H, I)** present the entire digestive tract, removed from the insect body where in panel **(H)** the insect ingested the blue dye and in panel **(I)** another insect did not ingest it, thus retaining its natural color. The gut consists of a clearly defined foregut (fg), midgut (mg), and hindgut (hg), with the proventriculus (pv) visible with a brown-hue marking the transition between the foregut (fg), and midgut (mg). The midgut is elongated and convoluted, while the hindgut appears wider and looped. Black dashes in panels **(H, I)** indicate boundaries separating the three gut regions. Panels **(J, K)** displays internal structures of the proventriculus (pv) **(J)** and the upper midgut (mg) **(K)** both dissected to expose their internal morphology with the luminal side facing up. Panel **(L)** provides a schematic diagram summarizing the gut anatomy, indicating the relative positions of the foregut (fg; blue), midgut (mg; brown), and hindgut (hg; green) within the insect’s body. Gut length (foregut to hindgut) was 1.63 ± 0.32 cm (mean ± SD; range: 1.2-2.7 cm; n = 23; males and females combined), with males 1.60 ± 0.20 cm (range: 1.2-1.8 cm; n = 9) and females 1.55 ± 0.24 cm (range: 1.3-2.0 cm; n = 12). Scale bars: 1 mm (all panels). *Abbreviations*: e, esophagus; t, trachea; fg, foregut; mg, midgut; hg, hindgut; mt, Malpighian tubules; pv, proventriculus.

To our knowledge, the gut anatomy of *D. abbreviatus* adults had not been previously characterized. Using a dye-based tracing approach, we visualized foregut, midgut, and hindgut structures and identified associated tissues such as tracheae and Malpighian tubules (**Figure 1**). After 24 hours of feeding Blue dye No.1 in agarose, we removed the insect’s head, which allowed us to locate the foregut stained with blue dye and revealed the tracheal system (**Figure 1A**). We then positioned the insect dorsally and removed the elytra (hardened forewings) and membranous wings, and exposed the dorsal abdominal cuticle. Careful removal of the cuticle revealed a network of tracheae running beneath it and above the abdominal organs (**Figure 1B**). Using tweezers, we removed the tracheae, which exposed the hindgut, clearly stained with blue dye and folded over the foregut and midgut (**Figures 1C–G**). We observed a network of Malpighian tubules surrounding the hindgut and converging near the hindgut–midgut junction (**Figure 1E**), similar in gross structure to the anterior midgut of the adult weevil *Epiphaneus malachiticus* Boheman (33). Small tracheae closely associated with the hindgut suggested localized gas exchange (**Figure 1E**) (53).

Once the entire digestive tract was removed, we clearly identified the foregut ending with the proventriculus, the midgut and the hindgut (**Figures 1H, I**). The proventriculus exhibited longitudinal ridges and chitinous masticatory teeth, resembling the muscular gizzard of other Coleoptera such as the coffee berry borer *Hypothenemus hampei* (Ferrari) and adult maize leaf weevil, *Tanymecus dilaticollis* (31, 54) (**Figure 1J**, **Supplementary Figure 1**). Just beyond the proventriculus, the midgut began with a wide region containing internal folds that may function as gastric caeca or crypts, increasing surface area for digestion and possibly housing symbiotic microbes (15) (**Figure 1K**). The gut length from foregut to hindgut was approximately 1.63 ± 0.32 cm (mean ± SD; range: 1.2–2.7 cm; n = 23; males and females combined). On average, male guts measured 1.60 ± 0.20 cm (range: 1.2–1.8 cm; n = 9), and female guts measured 1.55 ± 0.24 cm (range: 1.3–2.0 cm; n = 12). Overall, these findings provide the first anatomical characterization of the *D. abbreviatus* digestive system, highlighting structural adaptations that may contribute to its polyphagous feeding behavior (**Figure 1L**).

### 3.2 No sex differences in gut pH patterns of *D. abbreviatus* revealed by qualitative indicator dyes

**Figure 2:**
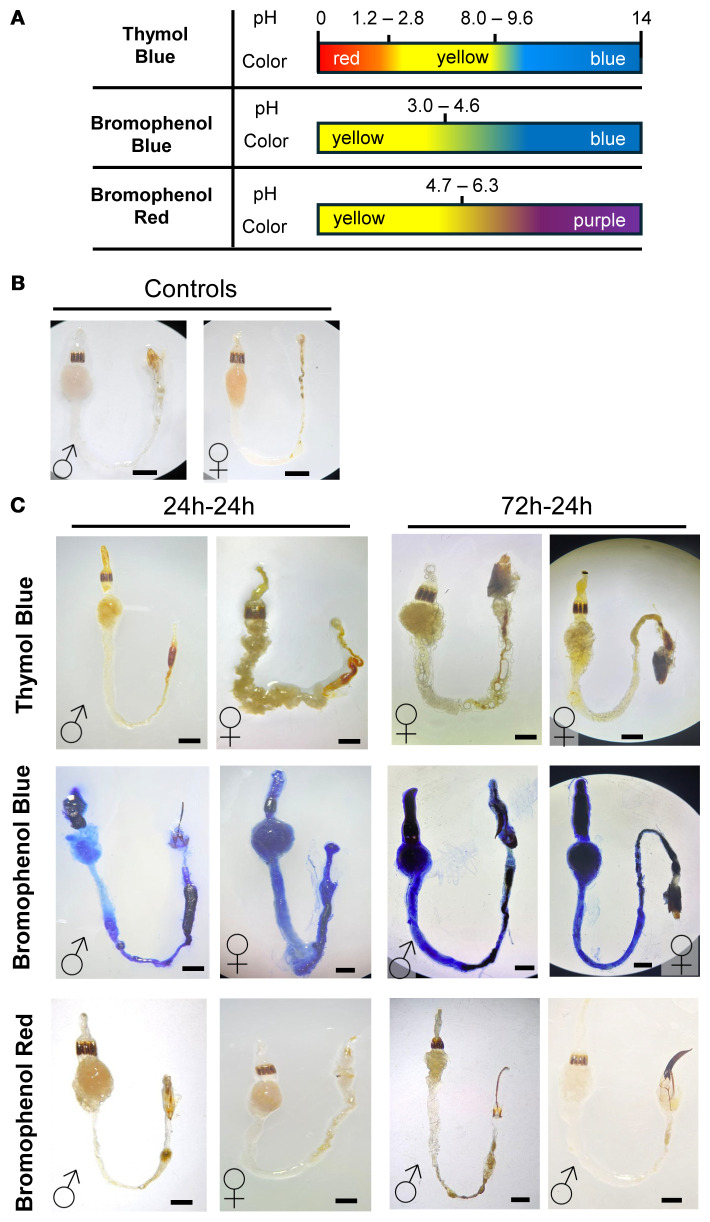
Qualitative gut pH assessment in male and female *D. abbreviatus* using colorimetric indicators. Schematic for the color transitions for three pH indicators: Thymol Blue, Bromophenol Blue, and Bromophenol Red **(A)**. Representative images of unstained guts in *D*. *abbreviatus* males (♂) and females (♀) **(B)**. Representative images of the male and female guts after feeding on each pH indicator for 24h-24h or 72h-24h (fasting-feeding paradigm). The top row shows dissected guts from insects that consumed Thymol Blue, the middle row shows guts from insects that consumed Bromophenol Blue, and the bottom row shows guts from insects that consumed Bromophenol Red. A total of three independent biological replicates were conducted, with 3–5 insects dissected per fasting or feeding condition **(C)**. All scale bars = 1mm.

As abovementioned, differences in pH have previously been noted between male and female insects, which is often due to differences in feeding habits (40). Evaluation of pH changes can be performed in various manners. To explore gut pH in *D. abbreviatus*, we employed the use of three pH indicators and compared patterns between males and females. Our results revealed no sex-specific differences, and staining patterns were consistent across treatments (**Figure 2**).

The indicators used (Thymol Blue, Bromophenol Blue and Bromophenol Red), and their pH transition ranges are indicated in **Figure 2A**. To ensure dye consumption, we tested three feeding paradigms after starving insects for 24, 48, or 72 hours, followed by 24 hours of feeding on the pH indicator/agarose. Staining patterns were consistent regardless of fasting duration (**Figure 2B**). For the 48-hour fasting group, only a limited number of replicates were available, but the staining patterns matched those of the other groups. Given the small sample size, these data are not presented as a separate panel in **Figure 2**.

Thymol Blue revealed a predominantly yellow gut, consistent with a broad acidic-to-neutral range, with occasional orange-red staining at the posterior hindgut, suggesting a more acidic region or fecal accumulation. Bromophenol Blue yielded strong blue coloration in the foregut and hindgut, indicating conditions above the acidic transition range. Bromophenol Red showed a yellow-to-pink/purple gradient, suggesting conditions spanning mildly acidic to near-neutral. The midgut displayed more pink/purple staining, while the foregut and hindgut remained mostly yellow, suggesting a gradient from mildly acidic to near-neutral pH.

Overall, these findings indicate subtle regional pH variation within the digestive tract. The foregut maintains a mildly acidic environment (approximately pH 4–5 based on qualitative ranges), with the midgut displaying a shift towards neutral or slightly alkaline conditions, and the hindgut exhibits variable pH levels, potentially influenced by fecal matter. Importantly, no sex-specific differences in gut pH were observed. These results provide a first step in understanding whether these slight regional pH differences contribute to digestion and shape distinct microbial niches in *D. abbreviatus*.

### 3.3 Gut microbiome diversity and taxonomic composition of *D. abbreviatus* does not differ across sex

**Figure 3:**
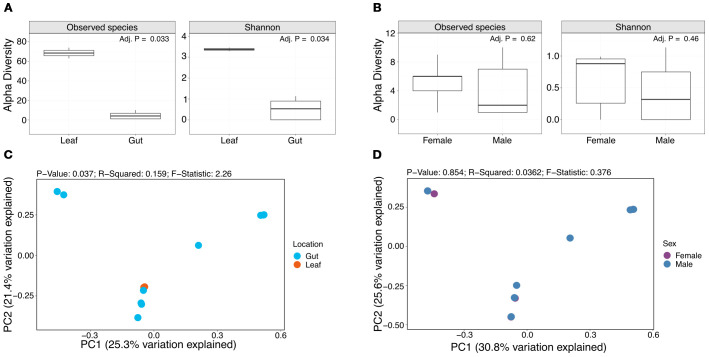
Alpha and beta diversity analyses comparing gut and leaf samples and sexes. Box plots show the number of observed species and Shannon diversity index of leaf and gut samples **(A)**, and female and male samples **(B)**. PCoA plots show beta diversity results assessed through Bray-Curtis distances for leaf and gut samples **(C)** and female and male samples **(D)**.

To investigate the gut microbiota diversity and composition of *D. abbreviatus*, we performed shotgun metagenomic analysis on fully dissected guts from male and female *D. abbreviatus* and of pooled lemon tree leaf samples from a farm in Puerto Rico (**Figure 3**). This study focused on the bacterial sequences obtained from the metagenomic analysis.

Alpha diversity was assessed through the number of observed species and Shannon diversity index (**Figure 3**). The median number of observed bacterial species in female and male samples was 6 and 2 species, respectively, and the median number of observed bacterial species in the leaf samples was 68. The median Shannon diversity was 0.88 and 0.32, for female and male samples, respectively, and 3.37 for the leaf samples. Wilcoxon analyses showed significant differences in the number of observed species (FDR-adjusted p-value = 0.033) and Shannon diversity (FDR-adjusted p-value = 0.034) between leaf and gut samples (**Figure 3A**), but not for female and male samples (**Figure 3B**). Beta diversity (Bray-Curtis dissimilarity) did not show clear clustering by sample type (i.e., leaf vs gut) (**Figure 3C**), or sex (**Figure D**).

**Figure 4:**
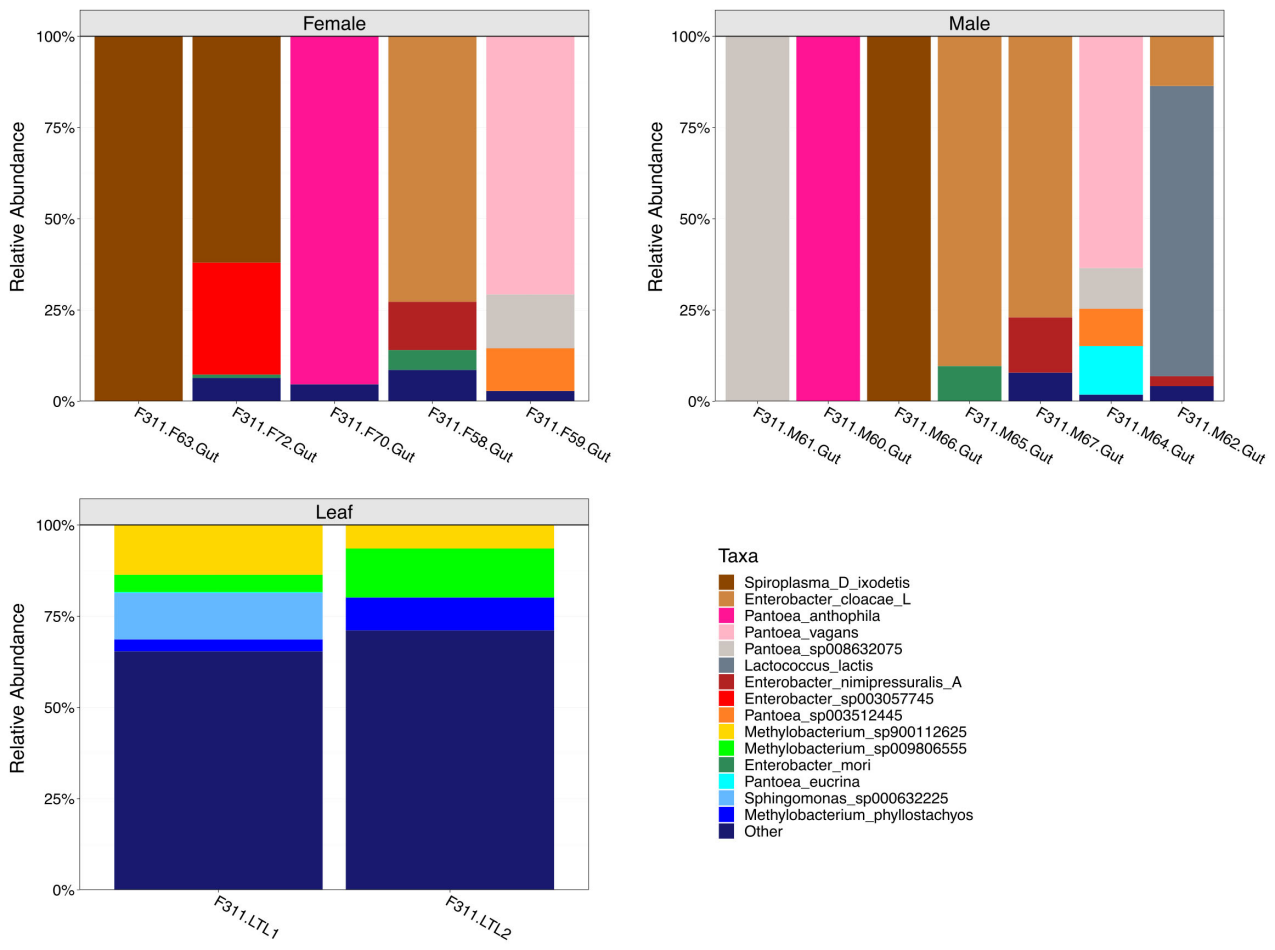
Taxonomic composition analyses of female, male and leaf samples. Stacked bar plots show the top 15 most abundant bacterial species in female, male and leaf samples. Taxa grouped as “Other” represent less abundant species.

The relative abundance of the top 15 most abundant bacterial species in female, male and leaf samples were visualized through stacked bar plots (**Figure 4**). Results showed individualized taxonomic profiles on a per sample basis. Particularly, results showed that some of the most abundant bacterial species in the gut samples belong to the *Spiroplasma* (*Spiroplasma D ixodetis*)*, Enterobacter* (e.g., *Enterobacter cloacae* and *Enterobacter mori*) and *Pantoea* (e.g., *Pantoea vagans* and *Pantoea anthophila*) genera. Interestingly, *Lactococcus lactis* was an abundant and uniquely identified species in one of the male gut samples. On the other hand, the pooled leaf samples (n = 2) were dominated by *Methylobacterium* spp. (**Figure 4**), which were largely absent from the guts.

**Figure 5:**
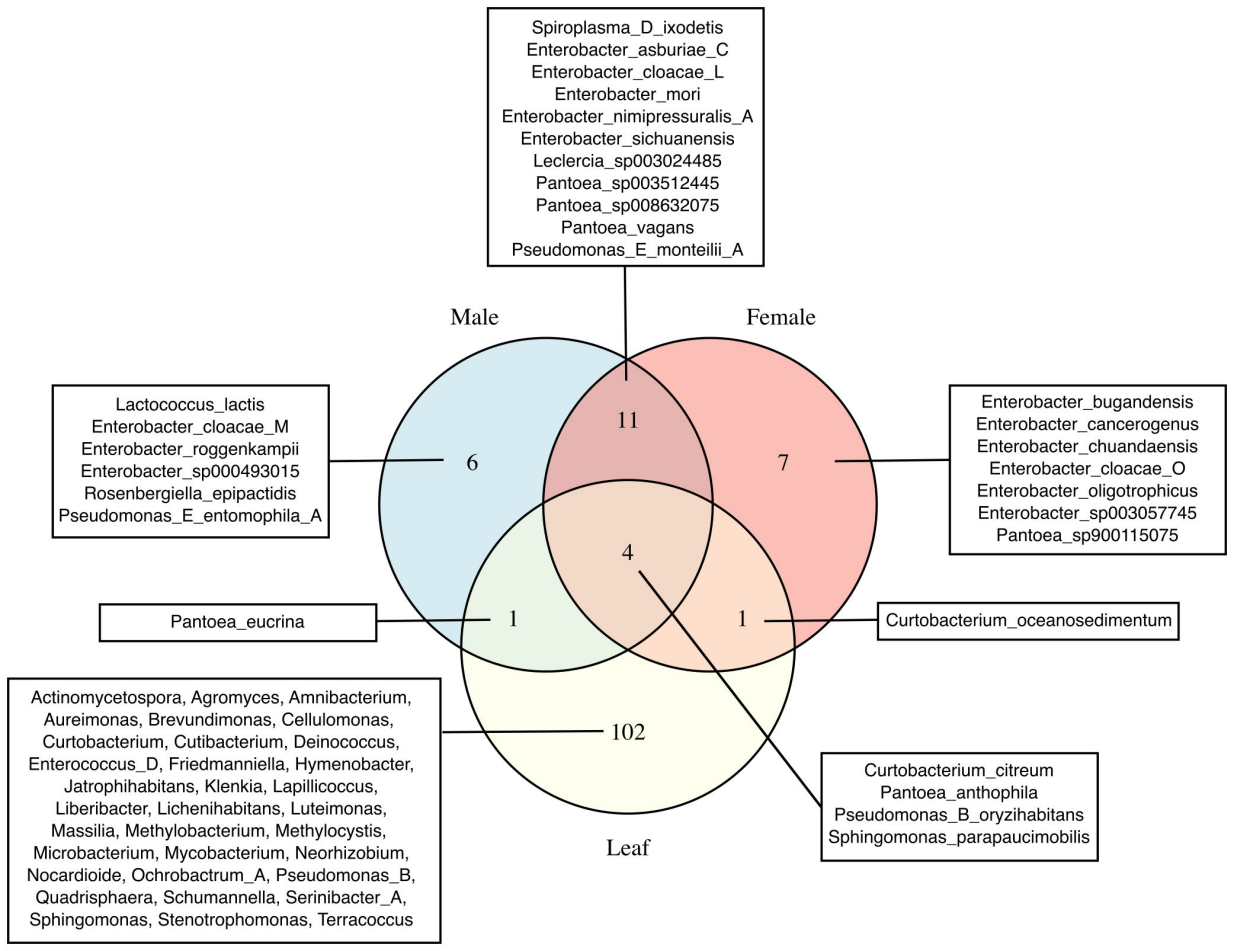
Unique and shared bacterial taxa in female, male, and leaf samples. Venn diagrams show the number of unique and shared bacterial taxa among female, male, and leaf samples. Species names are displayed for female and male samples, whereas genus names are shown for leaf samples due to the higher number of unique taxa in that group. GTDB suffixes (e.g., _A, _C, _M and _L) indicate *de novo* species clusters within a genus with no formal names and that are separate from the canonical species names.

The number of unique and shared bacterial species between female, male and leaf samples were visualized through Venn-diagrams (**Figure 5**). All sample types and individual insects within groups showed unique bacterial species. For instance, male samples were characterized by *Lactococcus lactis*, *Pseudomonas E entomophila A* and specific *Enterobacter* spp. that were not identified in the female samples. Similarly, the female samples were characterized mostly by *Enterobacter* spp., which were not identified in the male samples. In addition, over 100 unique bacterial species were identified in the leaf samples that could be categorized into over 30 different bacterial genera. Results also showed bacterial species that were present in both the gut and leaf samples. Some of these bacterial species included those from the *Curtobacterium, Pseudomonas, Pantoea* and *Spiroplasma* genera.

### 3.4 Gut bacterial co-correlations reveal potential sex-specific patterns

**Figure 6:**
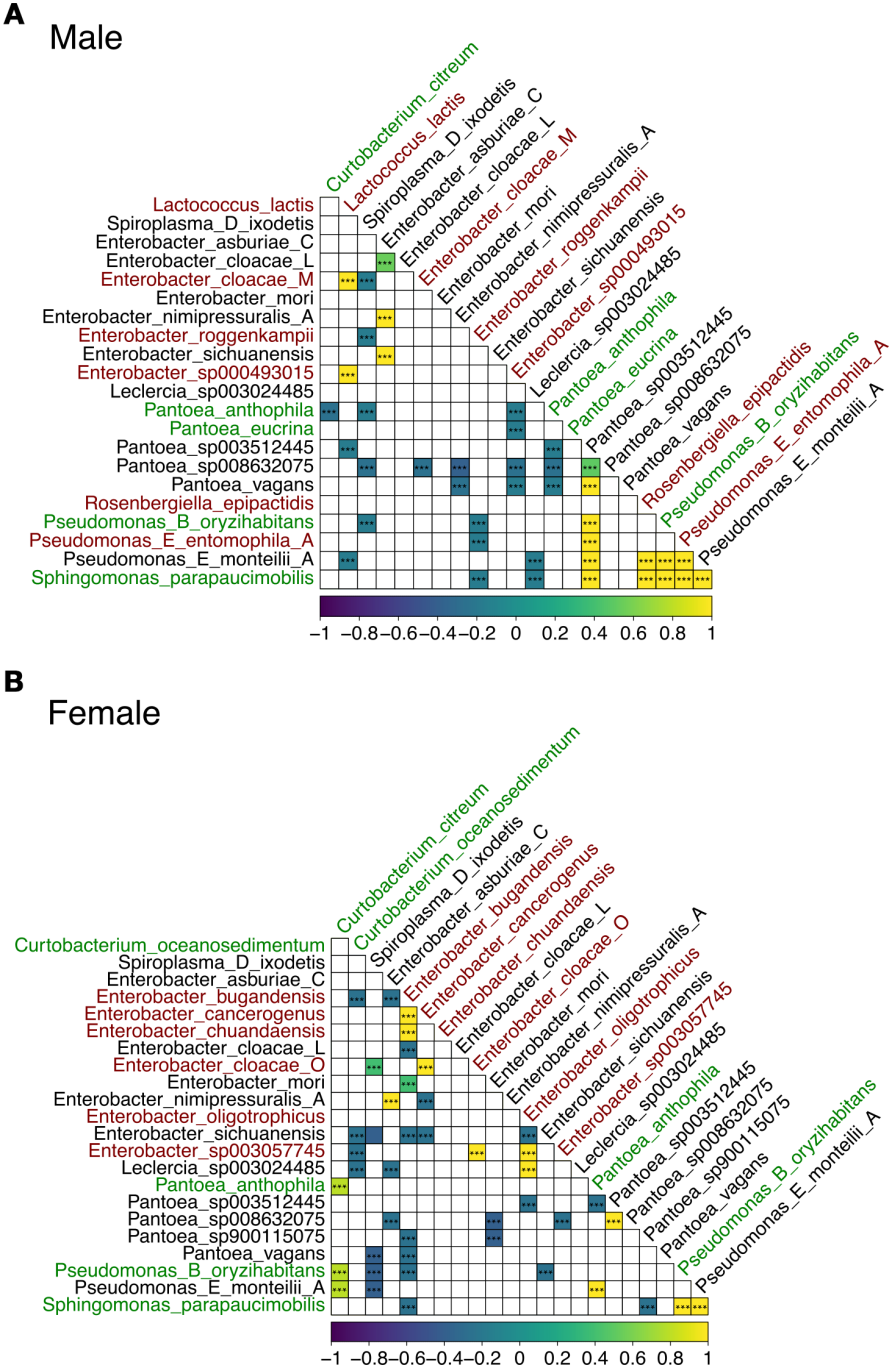
Spearman correlation heatmap of microbiome taxa abundances in male **(A)** and female **(B)** guts. Axes list the genus and species identified in the microbiome samples. Bacterial taxa unique to gut samples, shared between sexes, or also present in leaf samples are shown in red, black, and green, respectively. Each cell in the lower triangle of the matrix shows the correlation between the relative abundances of two taxa across samples. The color scale (bottom) indicates the Spearman correlation coefficient (ρ), with significance assessed after FDR-adjusted p-values. Yellow indicates positive correlations (co-occurrence), purple indicates negative correlations (mutual exclusion), and white/light colors indicate weak or no correlation (i.e., FDR-adjusted p-value < 0.05). GTDB suffixes (e.g., _A, _C, _M and _L) indicate *de novo* species clusters within a genus with no formal names and that are separate from the canonical species names.

The resulting correlogram of Spearman correlations revealed distinct patterns of association in male and female insects. Co-correlation analyses showed significant (FDR-adjusted p < 0.05) positive (yellow) and negative (purple) correlations between bacterial species in gut samples (**Figure 6**, **Supplementary Data Sheet 1**). Bacterial species names that are unique, shared or also present in leaf samples are shown in red, black and green text, respectively. Notably, certain bacterial species were identified in certain individuals in both male and female groups.

In males, we observed over 15 strong positive and over 20 negative bacterial species co-correlations (**Figure 6A**). For example, *Lactococcus lactis* was positively co-correlated with several *Enterobacter* spp. including *Enterobacter cloacae M*, which were all uniquely identified in the male gut samples. *Pantoea* sp003512445 was positively co-correlated with other *Pantoea* spp., *Rosenbergiella epipactidis* (uniquely identified in the male gut samples), several *Pseudomonas* spp., including *Pseudomonas B oryzihabitans* (also identified in the leaf samples)*, Pseudomonas E entomophila A* (uniquely identified in the male gut samples) and *Pseudomonas E monteilii A* (shared with female gut samples).

In females, notably, different co-correlation patterns were noted depending on the bacterial species (**Figure 6B**). For instance, over 15 and 25 significant positive and negative co-correlations were noted, respectively. *Enterobacter bugandensis*, uniquely identified in the female gut samples, was positively correlated with other uniquely identified species in the female gut including *Enterobacter cancerogenus* and *Enterobacter chuandaensis*, and negatively correlated with bacterial species also found in the male gut including *Enterobacter sichuanensis* and several *Pantoea* spp. *Enterobacter bugandensis* was also negatively correlated with bacterial species also identified in the leaf samples including *Pseudomonas B oryzihabitans* and *Sphingomonas parapaucimobilis*.

### 3.5 Unique and shared bacterial taxa identified across sample types and methods

As mentioned above, our initial shotgun metagenomics experiments, designed to explore gut microbiota differences across gut regions and sexes, were affected by human skin contamination. We then curated the dataset by identifying bacterial species known to be common on human skin and excluded or filtered them from subsequent analyses (see **Supplementary Table 3** for the list of removed taxa and **Supplementary Table 4** for the curated data). The shotgun metagenomics data presented in **Figures 3**–**6** were generated from insects collected and processed using enhanced precautions (see Materials and Methods), with DNA extraction and library preparation performed by Zymo Research. These samples did not exhibit detectable human skin bacterial contamination, supporting the validity of the filtering criteria applied to the dataset referred herein as the ‘Gut regions Shotgun Metagenomics’ dataset, indicating that the remaining taxa reflected the insect-associated microbiota. These findings also confirm the effectiveness of the improved collection and sterilization procedures in minimizing human-associated contamination.

While results did not show significant differences in alpha diversity between sexes (**Supplementary Figure 2A**), or gut region (**Supplementary Figure 2B**) at the genus level (likely due to diversity variability), there were trends of higher diversity values across specific comparisons. For instance, female samples showed higher Shannon diversity median values compared to male samples (**Supplementary Figure 2A**). Similarly, posterior gut or hindgut samples showed a trend of higher median values in terms of the number of observed genera and Shannon diversity index values (**Supplementary Figure 2B**). Interestingly, beta diversity results showed a degree of clustering based on sex, although sample variability was noticeable (**Supplementary Figure 2C**). Finally, taxonomic abundance analysis at the genus level showed sample-specific profiles, with *Lactobacillus* and *Pseudomonas* being among the most abundant across sexes and gut regions (**Supplementary Figure 2D**).

**Figure 7:**
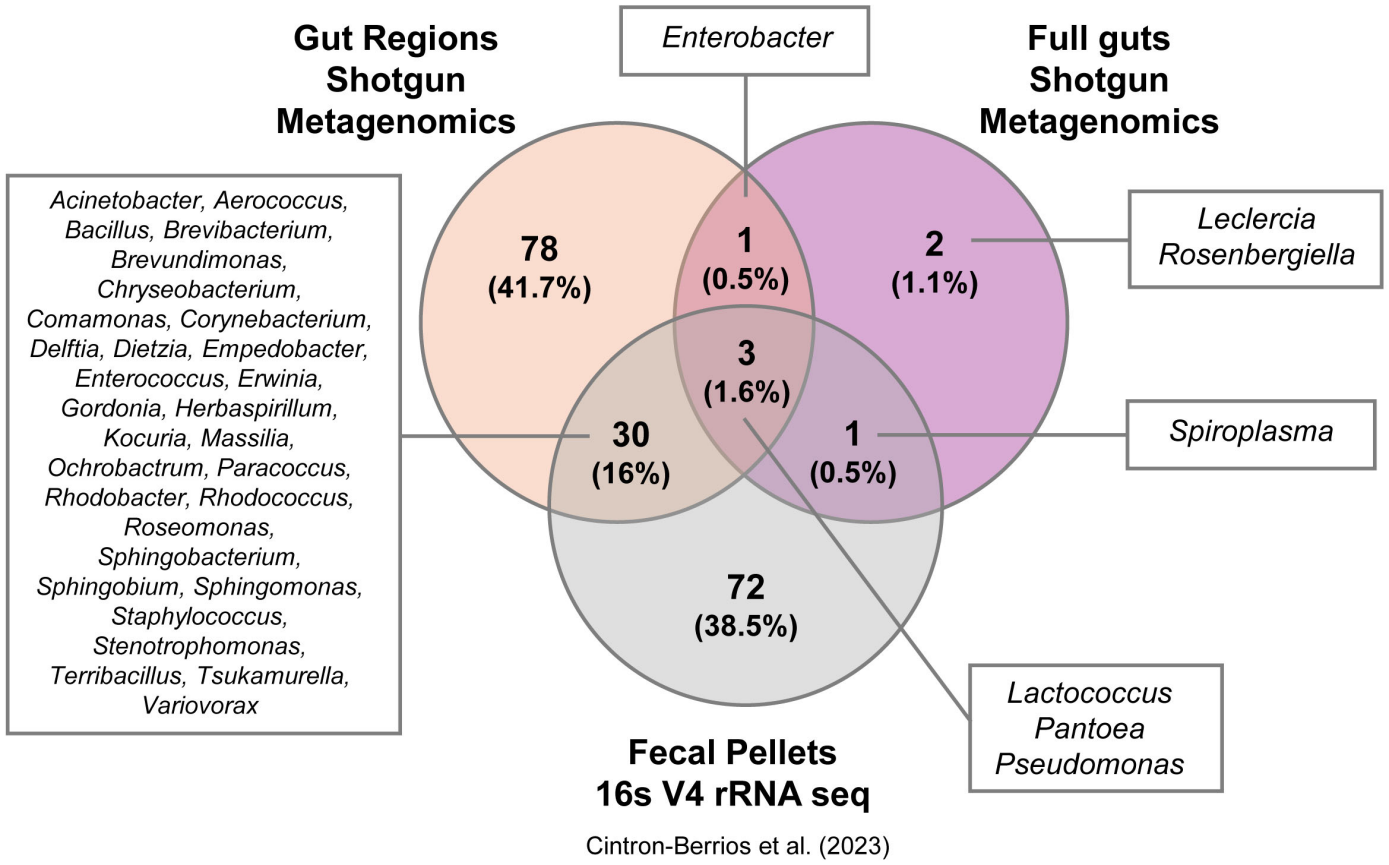
Unique and shared bacterial taxa detected by different methods. Venn diagram showing the number of unique and shared bacterial taxa identified using three methods on samples from *D. abbreviatus*: gut regions (shotgun metagenomics, Novogene, Inc.), full guts (shotgun metagenomics, Zymo Research), and fecal pellets (16S V4 rRNA sequencing, Mr. DNA Shallowater, Texas, U. S. A.; data from 28). Taxa names for each group are displayed in the boxes surrounding the diagram, with genus names shown for all groups.

To identify bacterial taxa that could represent potential symbionts and members of the core gut microbiota of adult *D. abbreviatus*, we compared the bacterial taxa detected using the three above mentioned approaches: (i) shotgun metagenomics of dissected full guts and gut regions (gDNA extracted in-house and sequenced by Novogene Inc.; this study (**Supplementary Figure 2**), (ii) shotgun metagenomics of full guts (gDNA extracted and sequenced by Zymo Research; this study (**Figures 3**–**6**), and (iii) 16S V4 rRNA gene sequencing of fecal pellets (gDNA extracted in-house and sequenced by MrDNA; data from (28). These datasets were also annotated using differing databases and classifiers due to the nature of the sequencing approach and commercial facility where sequencing was performed (**Supplementary Methods**). Overall, a total of 187 bacterial taxa were detected across all methods (**Figure 7**). The fecal pellet samples analyzed by 16S rRNA amplicon sequencing (V4) identified the largest proportion of taxa (72 unique taxa; 38.5%), including genera such as *Lactococcus*, *Pantoea*, and *Pseudomonas*. Shotgun metagenomics of gut regions revealed 78 unique taxa (41.7%), with representatives including *Acinetobacter*, *Aerococcus*, *Bacillus*, and *Enterococcus*. The full gut shotgun metagenomics dataset detected the fewest unique taxa (2 taxa; 1.1%), namely *Leclercia* and *Rosenbergiella*.

There was a notable overlap among the methods: 3 taxa (1.6%) were shared across all three datasets, while 30 taxa (16%) were shared between gut region metagenomics and fecal pellet 16S sequencing (**Figure 7**). Importantly, these 30 shared taxa with fecal pellets originate from the hindgut regions and not from midguts samples (**Supplementary Table 4**). Additionally, a single taxon (*Enterobacter*) was detected only in the gut region and full gut shotgun metagenomics studies. Another taxon (*Spiroplasma*) was only found in the full gut shotgun metagenomics and fecal pellets studies. Only 4 species were found to overlap between both shotgun metagenomics projects, namely *Enterobacter cloacae*, *Lactococcus lactis*, *Pantoea vagans* and *Pseudomonas monteilii* (**Supplementary Figure 3**). These results illustrate that the choice of collection method, sample type, database and classifier may substantially influence the diversity and composition of bacterial taxa detected in *D. abbreviatus*, where each method revealed distinct, yet overlapping subsets of the microbiome.

## 4 Discussion

This study provides the first comprehensive characterization of the gut microbial communities of *D. abbreviatus*, examining potential differences across gut regions and between sexes. We employed both qualitative and quantitative approaches, including colorimetry and shotgun metagenomic sequencing, to investigate these differences. Our results reveal that *D. abbreviatus* has a mildly acidic gut environment, with some regionally distinct microbial communities and limited overlap with dietary bacteria. In addition, while no statistically significant differences were noted in microbiome diversity and composition, some microbial associations appear to be sex-specific. Potential sex-specific differences in association with the microbiome have been identified previously in systems such as *D. melanogaster* and *A. gambiae*, but no studies have focused on *D. abbreviatus.* The lack of significance based on sex in our study may have been due to the individualized profiles noted across insects. Nevertheless, additional female and male samples in future work may serve as groundwork for developing sex-based control strategies for *D. abbreviatus*. For instance, female *D. abbreviatus* can lay up to 5,000 eggs, making potential mitigation strategies focus on females’ reproduction (8).

### 4.1 Gut pH and anatomy

Characterizing gut anatomy and pH is essential for understanding digestive processes and the environmental conditions that shape microbial communities potentially involved in digestion in insects (40, 45, 46). By feeding adult weevils Blue Dye No. 1 in agarose, we were able to distinguish the gut regions (foregut, midgut, and hindgut), as well as other associated tissues, such as tracheae and Malpighian tubules, and compare these structures to those of other beetles. Our data suggest that, similarly to other Coleoptera, *D. abbreviatus* has a muscular gizzard, equipped with sclerotized (hardened) structures that mechanically process food before it enters the midgut for digestion. Although we successfully obtained SEM images of the proventriculus, imaging the other gut tissues proved more challenging, and the protocol will require further optimization.

Our colorimetric analyses revealed a predominantly mildly acidic gut environment, consistent with a pH ranging from ~ 4–5, with slight variation among gut regions and no significant differences between sexes. Because these measurements were based on pH indicator dyes inspected by eye/photo, the results should be interpreted as qualitative ranges rather than precise quantitative values. This qualitative range is consistent with reports from other Coleopterans, such as the adult coffee berry borer (*Hypothenemus hampei* Ferrari) which has a gut pH ranging from 4.5 to 5.2 (55). In contrast, several other beetle species, including the larvae of the Colorado potato beetle (*Leptinotarsa decemlineata*), have more alkaline digestive tracts (i.e., pH 5.6–6.6) (56). Notably, the coffee berry borer is a specialist pest restricted to coffee, whereas the Colorado potato beetle is a polyphagous agricultural pest (56). Our finding that *D. abbreviatus*, a polyphagous agricultural pest, also exhibits a mildly acidic gut environment suggests that gut pH does not consistently align with host range breadth in Coleoptera. We note, however, that comparisons across studies may also reflect differences in life stage, as the acidic gut of adult coffee berry borers contrasts with the more alkaline gut reported for Colorado potato beetle larvae. Even so, gut pH likely plays an important role in shaping digestive physiology and microbial associations. However, quantitative pH measurements are needed to test this hypothesis.

This mildly acidic environment may facilitate digestion by enabling the activity of specific enzymes and aiding in the breakdown of plant materials in *D. abbreviatus*. Additionally, it may influence the gut microbiome composition by imposing pH-driven selective pressure, favoring acid-tolerant microbial taxa. Also, mild acidic pH could contribute to the degradation of ingested plant toxins, potentially enhancing *D. abbreviatus*’ ability to exploit a diverse range of host plants. This, in turn, may support its success as a polyphagous agricultural pest.

### 4.2 Gut microbial communities

Shotgun metagenomic sequencing of the full gut enabled us to characterize the gut microbiome of adult male and female *D. abbreviatus*. This approach posed several technical challenges, including field sample collection, in-house tissue homogenization, the low biomass of the gut samples for DNA extraction, and the limited similarity of gut bacterial species to those represented in current reference databases. Because insects were field-collected from lemon tree farms, we took care to control for external contaminants (e.g., human skin) by using a standardized dissection protocol and submitting samples to a commercial facility (Zymo Research) for DNA extraction. These precautions ensured that the microbial communities described herein reflect the gut environment and not external contaminants. This study, therefore, contributes both a methodological framework for microbiome analysis of field-collected insects, and novel insights into the microbiota of *D. abbreviatus*.

To determine the extent to which gut microbiota reflects diet versus host selection, we compared gut bacterial profiles to those from lemon tree leaves, as these were the insects’ food source at the moment of collection. We identified 108 bacterial species in leaf samples, but only six were also detected in gut samples (i.e., one species was detected in males only, one species was detected in females only, and four species were detected in both sexes). Despite active feeding on these plants, the minimal overlap between leaf and gut microbiota suggests strong host filtering or selection mechanisms shaping the gut community. Leaf samples (n = 2 composite, each containing ~ 30 leaves) were dominated by *Methylobacterium* spp., typical plant-associated bacteria, which were largely absent from gut samples (57).

The gut samples exhibited variation in microbial dominance, with some individuals showing strong skews toward a single bacterial species. While no statistically significant differences were noted based on gut region at the genus level, higher observed genera median values were noted in the hindguts or posterior guts compared to the full guts and midguts. Differences in bacterial populations were found along the gut regions, suggesting spatial variation in microbial composition. These findings support the idea that gut region-specific microenvironments influence microbial colonization.

Several bacterial taxa stood out as potentially important members of the *D. abbreviatus* gut microbiota. For instance, members of the *Enterobacter* genus, including *E. cloacae* and *E. mori*, were found in multiple samples and sometimes in high abundance. *Enterobacter cloacae* was previously identified as an endophyte in a medicinal plant (58), has been implicated in plant polymer degradation in termites and the red palm weevil (59–61), in nitrogen fixation in *Dacus* and *Bactrocera* fruit flies (62, 63), and in the production of trehalase used in trehalose hydrolysis (important carbon source for insects) (64). *Enterobacter mori*, though typically considered a plant pathogen, was isolated from the silkworm *Bombyx mori* and found to carry insecticidal genes (65). Neither species was detected in the leaf microbiota, reinforcing their likely host-association.

*Pantoea* species were another notable group identified in the gut microbiota of *D. abbreviatus*. *Pantoea* spp., which belong to the *Enterobacteriaceae* family, are facultative anaerobes frequently associated to plants as pathogens or beneficial bacteria (serving as biocontrol for other pathogens) depending on the context (66, 67). Some species were previously classified as *Erwinia* or *Enterobacter* (68). *Pantoea vagans* was detected in both male and female guts, but not in leaves, while *P. anthophila* was shared between guts and leaves. These species were originally isolated from diseased plants and flowering shrubs, respectively, (68), and have since been found as symbionts in phytophagous stinkbugs (family Pentatomidae) (69). Some *Pantoea* strains are obligate gut mutualists, contributing to host nutrition or defense. *Pantoea* species have been identified as obligatory mutualists in the midgut crypts of several phytophagous pentatomid stinkbugs, where their elimination leads to increased mortality and reduced fecundity (70–72). The presence of *Pantoea* spp. in the gut of *D. abbreviatus*, another polyphagous herbivorous beetle and crop pest, raises the possibility that similar mutualistic relationships in *D. abbreviatus* may exist, warranting further investigation into their functional role in host survival and adaptation.

Intriguingly, *L. lactis* was detected in one male gut sample in the main dataset, and in four additional samples across gut regions in the secondary dataset at the genus level. It was not found in feces, suggesting it may not be a transient dietary species. *Lactococcus lactis* is a lactic acid bacterium known for its acid tolerance and ability to metabolize sugars into lactic acid (73, 74). Its presence in the acidic gut environment of *D. abbreviatus* (approximately pH 4–5, based on qualitative ranges) is ecologically plausible since it has been found to be able to grow in pH between 4.8 – 6.5 (75). It was also recently identified as a core gut bacterium in a wood-boring beetle (76), highlighting its potential relevance in phytophagous insects.

*Spiroplasma ixodetis* was identified in several samples in high abundance. *Spiroplasma* spp. are bacteria without cell walls, known for diverse roles in insects, including reproductive manipulation, symbiosis, and pathogenicity (reviewed in (77)). Importantly, some *Spiroplasma* spp. have been shown to improve host fitness by providing protection against natural enemies such as nematodes, parasitoid wasps, and fungi. Among them, strains belonging to the *ixodetis* clade are among the most widespread in insects. Some aphid-infecting strains within this clade provide protection to pea aphids against the fungal pathogen *Pandora neoaphidis* (77–79). Notably, *Spiroplasma* strains infecting fruit flies *Drosophila atripex* and *Drosophila ananassae* fall into the *ixodetis* clade and are most closely related to *S. ixodetis* infecting ticks, as well as male-killing *Spiroplasma* strains found in a ladybird beetle (*Anisosticta novemdecimpunctata*) and butterfly (*Danaus chrysippus*) (80). The role of *Spiroplasma* in *D. abbreviatus* physiology remains to be explored, but its consistent presence in some individuals suggests a possible biological function.

*Pseudomonas* spp. are highly adaptable bacteria known for their metabolic diversity and ability to survive in a wide range of environments, including both aerobic and anaerobic conditions, in the presence of certain nutrients and electron acceptors (81). Members of this genus exhibit dual ecological roles, functioning as either beneficial endosymbionts or harmful pathogens. In insects, *Pseudomonas* can be transmitted via phoresy and may contribute to host defense, nutrient acquisition, and protection against microbial infections. Conversely, some species are known to produce toxins, form biofilms, and engage in quorum sensing, which can have pathogenic effects on the host (82). In our study, multiple *Pseudomonas* species were detected: *P. monteilii* was shared between sexes and detected in one hindgut sample, *P. entomophila* was observed only in males, and *P. oryzihabitans* was shared across all gut and leaf samples. The presence of *P. monteilli* in the hindgut may reflect adaptation to the low-oxygen, nutrient-rich conditions of the posterior gut, which can favor *Pseudomonadaceae* due to their versatile metabolic capabilities.

Although no significant differences in beta diversity and abundance were observed between male and female gut samples, co-correlation analysis revealed potential sex-specific bacterial interaction patterns. Specifically, results suggest possible context-dependent interactions that could be shaped by sex-specific physiology, immunity, or behavior. In addition, positive and negative co-correlations in taxa identified in the gut of *D. abbreviatus* opens the opportunity to determine mutualism, commensalism, cooperation and competition mechanisms. Here, positive correlations may suggest positive interactions (e.g., sharing resources), while negative correlations may indicate competition for resources, or inhibition of one species by another. Co-correlation analysis in *D. abbreviatus* can also help us understand which species co-exist through some of the mentioned mechanisms, or which species exclude each other.

### 4.3 Host-filtering of gut microbes

Across two shotgun metagenomics datasets, only four species were shared, namely *E. cloacae*, *L. lactis*, *P. vagans*, and *P. monteilii*, which were not detected in leaf samples. *Enterobacter cloacae*, *P. vagans*, and *P. monteilii* were detected in different hindgut or posterior samples from both sexes, suggesting they may be specialized for the hindgut environment. This spatial specificity supports the presence of structured microbial niches within the gut potentially shaped by factors such as regional pH and gut structure, which may act as ecological filters that influence microbial colonization. This interpretation aligns with simulations showing that phylosymbiosis (the tendency for closely related host species to have similar microbiota), often observed for gut microbiotas, can emerge from simple ecological filtering processes (e.g., gut pH variations), without requiring long-term coevolutionary relationships between hosts and microbes (83). Yet, ecological filtering and coevolution are not mutually exclusive, and the potential contribution of both processes cannot be ruled out in shaping the observed microbial patterns.

### 4.4 Limitations and future work

Our findings indicate that *D. abbreviatus* possesses a muscular, sclerotized gizzard and a predominantly mildly acidic gut environment (pH ~4–5), with no sex-specific differences in gut pH patterns, based on qualitative assessments with colorimetric indicators. Because these measures are qualitative, they should be interpreted with caution. Future studies could focus on obtaining quantitative pH measures by employing pH-sensitive microelectrodes or ratiometric fluorophores combined with imaging to explore finer regional pH gradients. These quantitative approaches will help us validate our qualitative measures on the gut pH. Moreover, integrating pH data with redox potential measurements would help define the physicochemical niches that structure microbial communities along the gut.

Our gut microbial community structure analyses relied on relative abundances, which, while widely used in microbiome studies, must be interpreted with caution given the compositional nature of such data (84, 85). One important limitation of our study is that relative abundance alone does not provide direct information about absolute microbial biomass. For instance, in some individuals, *Spiroplasma* appeared to dominate the gut community. This raises the possibility that the observed dominance could reflect either genuinely high *Spiroplasma* titers, which is well documented in insects, including weevils and drosophilids (86, 87), or low overall bacterial load. While composition-aware analytical methods can help mitigate biases inherent to microbiome data, absolute quantification approaches (e.g., qPCR with standards or colony-forming unit counts) would provide stronger evidence and remain an important direction for future work. In addition, selection of additional female and male samples in future work may serve as groundwork for developing potential sex-based control strategies for *D. abbreviatus*.

Finally, future research should prioritize the functional role of *D. abbreviatus* gut microbiota. The recent sequencing of *D. abbreviatus* genome revealed gene families encoding plant cell wall–degrading enzymes (PCWDEs) and invertases, including carbohydrate esterases, polysaccharide lyases, and glycoside hydrolases (27). Similar findings in other beetle species (Coleoptera) suggest that PCWDEs were acquired through convergent horizontal gene transfer from bacteria and fungi, enabling symbiont-independent digestion of plant material (88). Although *D. abbreviatus* encodes several PCWDEs and invertases, the consistent presence of microbial taxa with potential enzymatic plant tissue degradation capacity suggests that symbionts may complement host-derived digestive functions, particularly under dietary or environmental stress. One possibility that deserves further exploration, is that some gut-associated microbes may represent evolutionary remnants of ancestral symbioses that facilitated horizontal gene transfer, while others may have co-evolved to optimize digestion alongside host-derived enzymes. Also, further characterization of the gut physicochemical environment could help determine whether specific gut regions favor microbial activity, while others rely more heavily on host enzymatic processes.

### 4.5 Conclusions

Overall, our data suggest that the gut microbiota of *D. abbreviatus* is mostly shaped by host physiology and gut environment than by diet alone. While further sampling is needed to determine whether certain taxa constitute a core microbiome, consistent detection of certain species across individuals and datasets suggest non-random, and potentially functional associations. Future studies should explore bacterial function through the characterization of bacterial isolates, and the addition of several ‘omics such as transcriptomics, or metabolomics. In addition, since the insect gut microbiota encompasses more than bacteria, future research should also investigate the structure and potential roles of fungi, viruses, and other microorganisms in shaping this complex community. While this study focuses on adult *D. abbreviatus*, it would be valuable to characterize the gut microbiota across developmental stages, particularly in larvae, which occupy different ecological niches and have distinct feeding behaviors and gut physiology. Given that larval stages can last up to 15 months and feed on structural plant roots, their gut microbial communities may differ substantially from those of adults and contribute uniquely to host nutrition or development. Together, these efforts may help uncover microbial traits that contribute to host adaptation and could be of interest to novel microbiome-based approaches to pest management.

## Data Availability

The datasets generated and analyzed for this study can be found in the NCBI under PRJNA1297587 (metagenomics of full guts) and under PRJNA1234694 (metagenomics of gut regions).
