# Supplementary material for: Gut microbial community structure of the adult citrus root weevil Diaprepes abbreviatus
Source: Front Insect Sci. 2025 Oct 28;5:1676003. doi: 10.3389/finsc.2025.1676003 (PMC12604102; doi:10.3389/finsc.2025.1676003)
Supplement: Supplementary file 5 [file DataSheet1.pdf]

Supplementary Material

1 Supplementary Methods

1.1 Insect Collection and Dissection

Insects used for experiments characterizing bacteria from different gut regions were collected during June and July 2023 from Finca La Tres Once, Florida, Puerto Rico (18.3722° N, 66.5328° W). Insect collection was done without gloves, placed in a plastic container with holes to allow air transfer, and brought alive to the lab. Twenty-four hours later insects were surface sterilized using 70% ethanol for 30 seconds, followed by two 10-second washes of sterile PBS. Dissection was done using axenic techniques. Insect gut dissection process started with surface sterilization: the legs and heads were removed, along with chitin and wings, and the remaining body was thoroughly sterilized using 70% ethanol for 30s under agitation. Following two sterile PBS washes, insects were placed on a sterile petri dish. Using sterilized tweezers, we opened the thorax and abdomen and added 20 µl of TRIzol LS Reagent™ (Invitrogen, Carlsbad CA, USA, Cat. No. A33252) to the exposed cavity for one minute. The entire gut was removed from the rest of the body and placed directly on 200 µl TRIzol, then manually homogenized with a sterile disposable pestle (Fisherbrand, Waltham, MA, USA, Cat. No. 12-141-368).

For the samples containing specific gut regions, we sterilized the insects as explained above and after dissection the guts were divided into three gut regions: foregut, midgut and hindgut gut. These regions were carefully separated using tweezers, with 3 regions pooled per tube and manually homogenized. Finally, to all tubes, we added 800 µl of TRIzol, bringing a total volume of 1000 µl. Prepared samples were stored at –20°C, as suggested by the manufacturer, until DNA extraction could be performed following the manufacturer's protocol.

1.2 DNA extraction, and Purification (done *in-house*)

For samples characterizing bacteria in distinct gut regions, total DNA was extracted from insect full gut or regions using the TRIzol Reagent Experimental Protocol for DNA isolation (Invitrogen Corporation, Carlsbad, CA, USA) and subsequently purified through the Ethanol Precipitation protocol (Barrick Lab, The University of Texas at Austin, TX, USA). DNA concentrations and purity were measured utilizing Qubit® dsDNA High Sensitivity Assay Kit (Life Technologies, Carlsbad, CA, USA), and Nanodrop Spectrophotometer (ThermoFisher Scientific,Waltham, MA, USA), while DNA quality was determined using a 1% agarose gel electrophoresis.

1.3 Shotgun Sequencing and Quality Filtering (outsourced to Novogene)

Purified DNA samples were sent to Novogene Corporation Inc. (Sacramento, CA, USA) for library preparation and shotgun sequencing on an Illumina Novaseq6000 platform using a 2 × 150 bp configuration. To detect potential DNA degradation and contamination, DNA sample quality was monitored on 1% agarose gels. To evaluate DNA purity indicators (OD260/OD280, OD260/OD230), the NanoPhotometer® spectrophotometer was used (IMPLEN, CA, USA).

DNA concentration was measured using the Qubit®dsDNA Assay Kit in Qubit® 2.0 Fluorometer (Life Technologies, CA, USA). Only high-quality samples meeting the stringent criteria–

–DNA samples with OD values between 1.8 and 2.0 and concentrations above 1μg were selected for library construction. For each sample, a standardized input of 1μg of DNA was used. Libraries were prepared using the NEBNext® Ultra™ DNA Library Prep Kit for Illumina (NEB, USA), according to the manufacturer's protocol, with unique index codes added to each sample to facilitate downstream analysis.

The sequencing data underwent comprehensive processing through Novogene Corporation Inc., (Sacramento, CA, USA) analysis pipeline. Initial quality assessment of raw data was performed using readfq and fqcheck software. Host DNA sequences were then filtered out using soap 2.21 and bwa-0.7.10. This quality control and host filtering process yielded clean, high-quality data optimized for subsequent microbiome analyses.

1.4 Metagenome Annotations

After the initial quality filtering, metagenomic reads were annotated using Kraken2 (Galaxy Version 2.1.3) as described by Lu and Salzberg (2020). Briefly, paired-end reads were uploaded and annotated using the Prebuilt RefSeq: Standard Full (version from 2022-06-07) database, with a confidence value of 0.8. Minimum Base Quality and Minimum Hit Groups parameters were set to default (0 and 2, respectively). The output from the annotation was then used as input for the Kraken taxonomic report tool (Galaxy Version 0.0.3+galaxy1) using default parameters and the Kraken bacterial database. Outputs were then downloaded and analyzed using RStudio (v4.2.2). Kraken2 outputs were filtered to keep bacteria with classification at the species level, and species level classifications were collapsed to the genus level. Tables were further processed to filter taxa with relative abundances < 0.01% using the "Fraction.Filter" function from the R package MicrobeR (https://rdrr.io/github/jbisanz/MicrobeR/man/Fraction.Filter.html).

1.5 Diversity and Taxonomy Analyses

Bacterial alpha diversity (within sample diversity), including observed genera and species, and Shannon diversity and Inverse Simpson Index at the genus and species levels were calculated from the rarefied taxonomy tables (1,400 sequences) and using the phyloseq (v1.42.0) package in RStudio (v4.2.2). These diversity metrics were then visualized through boxplots constructed using ggplot (v3.4.0). Paired Wilcoxon analyses with False Discovery Rate (FDR) correction were performed. The rarefied phyloseq OTU table was then exported to tabular format for beta diversity and taxonomy analyses. Beta diversity (between sample diversity) was determined using Centre-log-ratios (CLR) transformation of the sequence counts using the rarefied table. Aitchison distances (the Euclidean distance between the CLR-transformed counts) were then calculated using the ALDEx2 package (https://www.bioconductor.org/packages/devel/bioc/vignettes/ALDEx2/inst/doc/ALDEx2_vignette.html) in R. Biplots were then constructed from the calculated Aitchison distances using ggplot2 (v3.4.0). Relative abundances of the top 20 most abundant bacterial genera and species were visualized through stacked plots using the MicrobeR package (https://rdrr.io/github/jbisanz/MicrobeR/man/Microbiome.Barplot. html) in R and the rarefied taxonomy table.

1.6 Scanning Electron Microscopy imaging

To visualize the morphology and characteristics of the adult *D. abbreviatus* proventriculus, we used a JEOL 6480LV scanning electron microscope (SEM) in low-vacuum mode (Molecular Sciences Research Center, San Juan, P.R.). The gut of *D. abbreviatus* was dissected and divided into three

portions (foregut, midgut, and hindgut). Each region was filleted and fixed by immersion for 2 hours in a solution of 2% paraformaldehyde and 2% glutaraldehyde. The tissue was then washed with distilled water, post-fixed with osmium tetroxide for 20 minutes, washed again with distilled water, dehydrated in absolute ethanol, and placed in a desiccator for 24 hours.

The samples were taken to the University of Puerto Rico (UPR) Molecular Sciences Research Center, where they were placed in microporous capsules (30 μm), immersed in absolute ethanol, and dried. Finally, the dried tissue was mounted on SEM stubs using gold conductive paint and examined under the JEOL 6480LV SEM in low-vacuum mode (Vilanova et al., 2016). Images of the proventriculus were captured at different magnifications (30×, 50×, 160×, 600×, 1,400×, and 3,300×). Notably, only the integrity of the proventriculus was preserved for SEM imaging, whereas the foregut and hindgut regions were too fragile to withstand processing and will need additional optimization.

2 Supplementary Figures and Tables

2.1 Supplementary Tables

List of Supplementary Tables:

Supplementary_Table _1_Zymo_ReadProcessingSummaryTable

Supplementary_Table _2_Zymo_species_allSamples

Supplementary_Table_3_Novogene_Species_Eliminated

Supplementary_Table_4_Novogene_Species_Raw_and_Filtered

2.2 Supplementary Figures

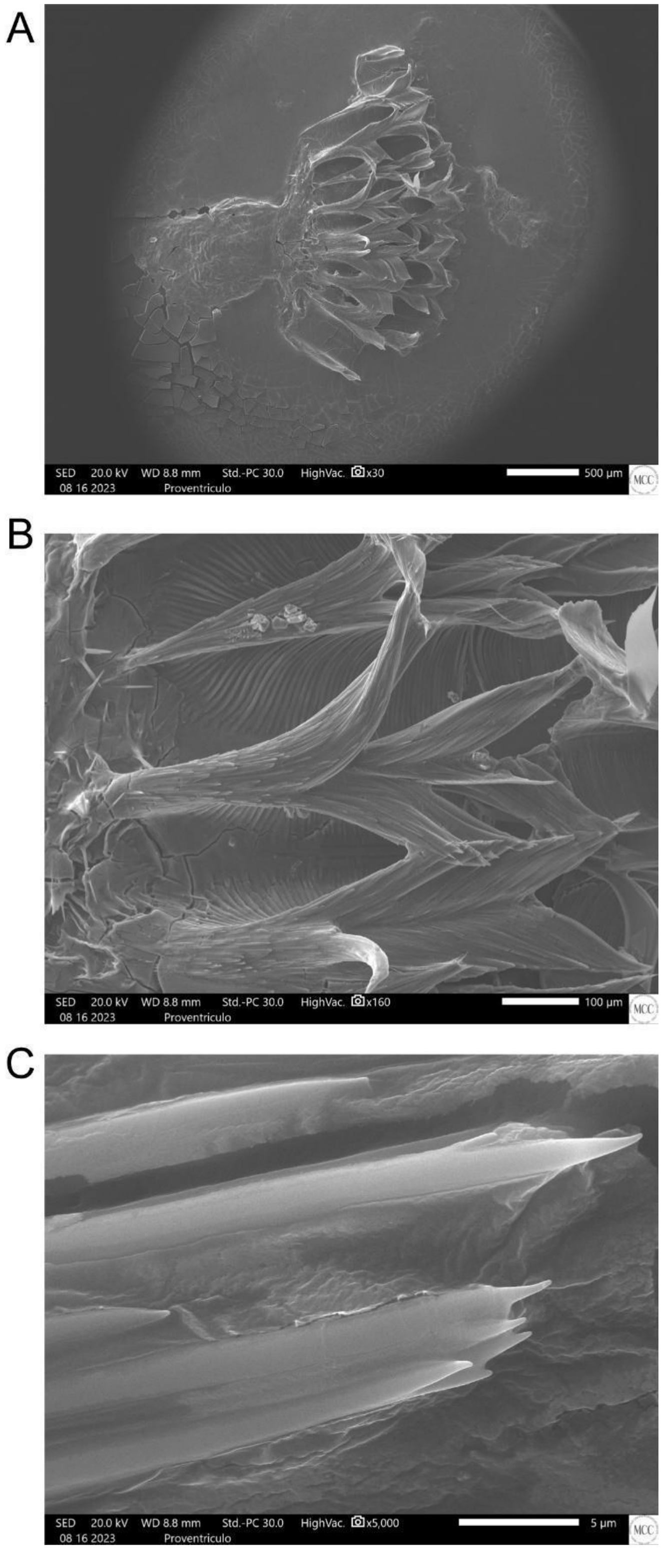

Supplementary Figure 1. Scanning Electron Microscopy (SEM) images of the *D. abbreviatus* proventriculus. Scale bars: 500 µm (**A**), 100 µm (**B**), and 5 µm (**C**).

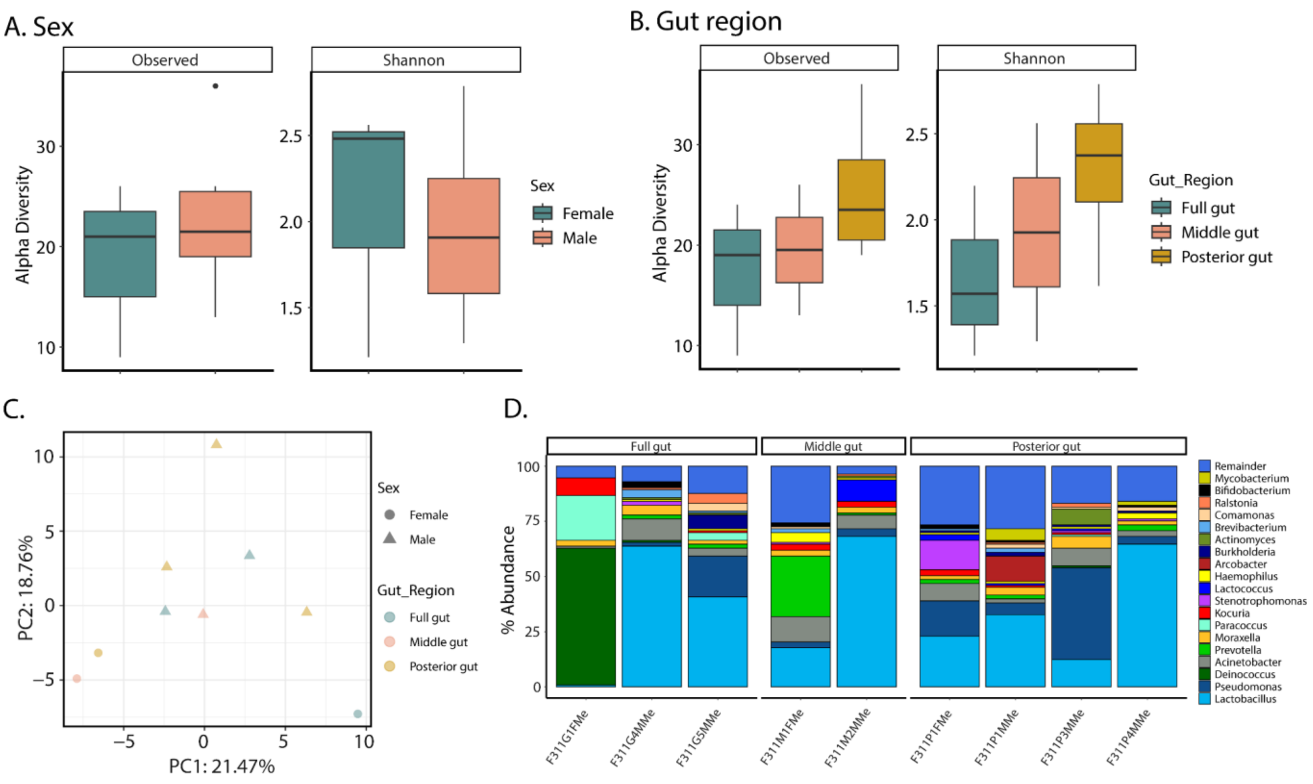

Supplementary Figure 2. Gut microbiota diversity and composition across sex and gut regions. Bacterial alpha diversity at the genus level, shown as boxplots of observed richness and Shannon diversity indices by sex (**A**) and gut region (**B**). Colors indicate sex (A) or gut region (B). Bacterial beta diversity based on Aitchison distances at the genus level. Points are colored by gut region and shaped by sex (**C**). Taxonomic composition of bacterial communities. Stacked bar plots show the relative abundance of the top 20 most abundant bacterial genera across samples. Samples are grouped by gut region (full, middle, posterior) and labeled below each bar according to farm of origin (F311 = Finca La Tres Once, Florida, Puerto Rico), gut region (G = full gut, M = midgut, P = posterior hindgut), sample number, and sex (F = female, M = male) (**D**).

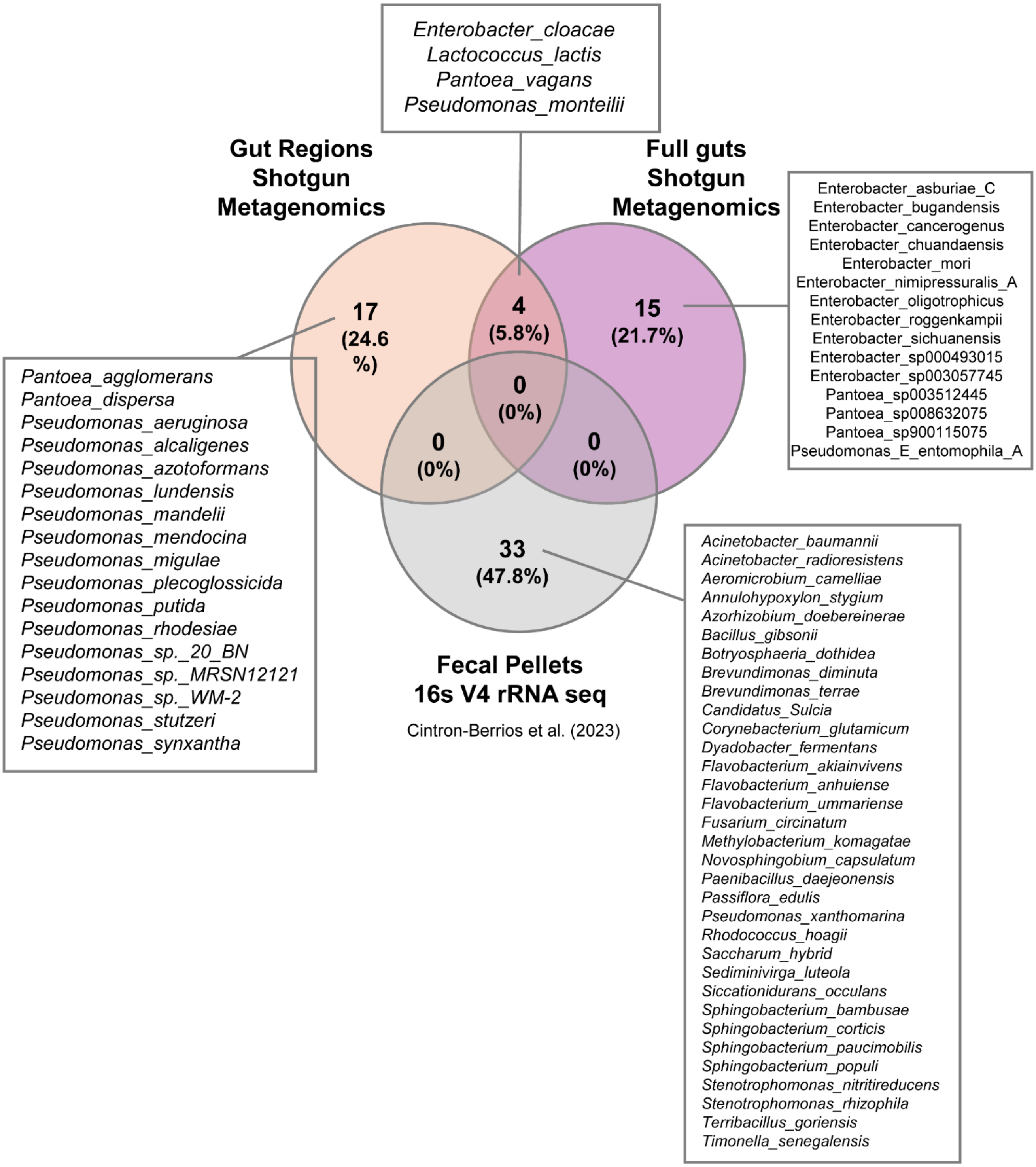

Supplementary Figure 3. Unique and shared bacterial taxa detected by different methods. Venn diagram showing the number of unique and shared bacterial species identified using three methods on samples from *D. abbreviatus*: gut regions (shotgun metagenomics, Novogene, Inc.), full guts (shotgun metagenomics, Zymo Research), and fecal pellets (16S V4 rRNA sequencing, Mr. DNA Shallowater, Texas, U. S. A.; data from Cintron-Berrios et al., 2023). Taxa names for each group are displayed in the boxes surrounding the diagram, with species names shown for all groups.
